# Altered hippocampal morphology and its association with gait impairment in idiopathic normal-pressure hydrocephalus

**DOI:** 10.3389/fneur.2026.1914240

**Published:** 2026-10-13

**Authors:** Jaeil Kim, Shin Young Jeong, Sang-Woo Lee, Uicheul Yoon, Kyunghun Kang

**Affiliations:** 1 School of Computer Science and Engineering, Kyungpook National University, Daegu, Republic of Korea; 2 Department of Nuclear Medicine, School of Medicine, Kyungpook National University, Daegu, Republic of Korea; 3 Department of Biomedical Engineering, Daegu Catholic University, Gyeongsan-si, Republic of Korea; 4 Department of Neurology, School of Medicine, Kyungpook National University, Daegu, Republic of Korea

**Keywords:** Alzheimer’s disease, gait, hippocampus, idiopathic normal-pressure hydrocephalus, magnetic resonance imaging

## Abstract

Background and purpose: This study aimed to characterize structural alterations of the hippocampus associated with idiopathic normal-pressure hydrocephalus (INPH), and to explore their relationship with gait disturbances in affected patients.

**Methods:**

A total of 48 patients with INPH and 25 β-amyloid–negative healthy controls underwent MRI-based, vertex-wise three-dimensional hippocampal shape analysis. Additionally, all participants received 18F-florbetaben positron emission tomography to assess amyloid status.

**Results:**

Hippocampal shape analysis revealed marked bilateral deformation in the INPH group compared to controls. Both hippocampi exhibited elongation and narrowing, with the middle and posterior regions showing upward curvature, indicating vertical displacement from the hippocampal body to the tail. The normalized hippocampal volume was significantly smaller in INPH patients. No substantial volumetric or morphological differences were observed between amyloid-positive and amyloid-negative individuals within the INPH group. Notably, localized shape deformities correlated with performance on the Timed Up and Go and 10-meter walk tests, more prominently in the right hippocampus. Bilateral inward deformations at the superomedial tail and inferior body of the hippocampus were consistently linked to worse gait performance. Furthermore, smaller right normalized hippocampal volumes were negatively associated with gait test outcomes.

**Conclusion:**

These findings indicate that INPH is associated with characteristic hippocampal shape abnormalities. The observed associations between hippocampal morphology and gait disturbances suggest that the hippocampus may contribute to gait and postural control in INPH.

## Introduction

Idiopathic normal-pressure hydrocephalus (INPH) is a treatable neurological condition typified by normal cerebrospinal fluid (CSF) pressure at lumbar puncture, dilatation of the ventricles, and symptoms of gait disturbance, urinary dysfunction, and cognitive impairment (1). While INPH patients can exhibit any of the described symptoms, gait disturbance is clinically the most relevant (1). The CSF tap test (CSFTT) is the most common way to diagnose INPH. Under the Japanese guidelines, clinical improvement following the CSFTT improves INPH diagnostic confidence from possible to probable (2).

β-Amyloid (Aβ) deposition is a central marker of Alzheimer’s disease (AD). According to the amyloid cascade hypothesis, it is believed to be the primary cause in the sequence of events that ultimately results in neurodegeneration and cognitive decline (3–5). Earlier pathologic changes in Aβ can cause later downstream neurodegeneration, subsequently causing a decrease in hippocampus size along with adverse clinical symptoms (6, 7). Since INPH occurs in elderly individuals, concomitant AD is also possible (8, 9). Glymphatic function in INPH can be altered, which can be a mechanism that explains the comorbidity between INPH and AD (10). In addition, INPH is considered a risk factor for developing AD (11).

Atrophy of the hippocampus is a central feature in AD patients (12). Hippocampal atrophy is understood to be a key biomarker in AD imaging (13). Studies on hippocampal volume in INPH patients have also been conducted; however, there has been only moderate agreement in the findings. While some studies on INPH patients have reported no hippocampal atrophy (14, 15), most studies have reported a decrease in hippocampal volume (16–20). Although these terms are sometimes used interchangeably in the literature, hippocampal volume reduction may result from neurodegenerative tissue loss, hydrocephalus-related mechanical deformation, or both. Consequently, whether the reported hippocampal abnormalities in INPH primarily represent true neurodegenerative atrophy, hydrocephalus-related deformation, or a combination of both remains unclear. Furthermore, given that concomitant amyloid pathology is not uncommon in patients with INPH, distinguishing hydrocephalus-related hippocampal alterations from amyloid-associated neurodegeneration remains an important unresolved issue.

To our knowledge, three-dimensional surface-based morphometric analysis has not been applied to characterize hippocampal shape alterations in INPH. The purpose of this study was to determine INPH-related hippocampus structural changes and investigate the relationship between hippocampal structural changes and gait disturbance in INPH patients. We hypothesized that hippocampal morphological abnormalities in INPH would be largely independent of amyloid pathology and would be associated with gait impairment.

## Methods

### Participants

INPH patients admitted to the Center for Neurodegenerative Diseases of Kyungpook National University Chilgok Hospital, South Korea, from June 2018 to March 2021 were prospectively enrolled in this study. INPH diagnosis was ascertained with the Relkin criteria (1). This involved extracting 30–50 mL of CSF from a patient with a lumbar CSFTT. Following the CSFTT, INPH patients were examined again using the Timed Up and Go Test (TUG), the Korean-Mini Mental State Examination (K-MMSE), and the INPH Grading Scale (INPHGS). Gait changes were ascertained many times over a 7-day period following the CSFTT, and symptomatic changes were measured at the end of the period. The responses were analyzed using 3 scales (2). Finally, INPH patients demonstrating a positive CSFTT response were added to the study (2). Regarding ^18^F-florbetaben PET, the brain amyloid-β plaque load (BAPL) score was measured according to a visual ratings system (21). In this case, a BAPL score of 1 was considered as β-amyloid negative, and BAPL scores of 2 and 3 were considered as amyloid-β positive.

Healthy controls were randomly picked from our general cohort of patients and were age and sex matched to the INPH patients. The criteria for determining healthy controls were as follows: (1) no current neurological, systemic, or psychiatric disorders; (2) normal neurological status; and (3) functional independence. Healthy controls determined to be β-amyloid negative by ^18^F-florbetaben were included in the study. Further, the cognitive ability of healthy controls was also analyzed using the K-MMSE.

### MRI imaging acquisition

MRI data were taken with a 3.0 T system (GE Discovery MR750, GE Healthcare). Three-dimensional sagittal, T1-weighted, and inversion-recovery fast spoiled gradient echo (IR-FSPGR) MRI images of the whole head (sagittal slice thickness 1.0 mm, no gap, TE = 3.2 ms, TR = 8.2 ms, field of view = 240 mm, matrix size 256 × 256 pixels, and flip angle 12°) were taken.

### Image analysis

#### Volumetric analysis

For analysis of hippocampal volume, pipeline image processing steps were employed. MRI in the native space was normalized spatially to a stereotaxic space and were corrected for intensity non-uniformity artifacts (22, 23). A multiscale nonlinear registration was employed to normalize the skull-stripped MR images using a brain extraction tool and to provide *a priori* information, i.e., tissue probability maps for further tissue classification with a neural network classifier (24). A trimmed minimum covariance determinant method was used to determine and correct partial volume errors that use MRI intensity mixing at tissue interfaces as a result of finite resolution of the imaging device (25). A hierarchical multi-scale non-linear fitting algorithm was then used to determine a 3D deformation vector field that maps the International Consortium for Brain Mapping (ICBM) 152 symmetric template onto the individual brain volume (22). Next, the resulting vector field was applied to transform the hippocampus atlas (26). Hippocampal volumes were determined using a graph-cuts algorithm (27). First, the atlas-based segmentation result was used to determine the first hippocampal region, which would be used *a priori* information for graph-cuts. The definition of initial seeds was additionally determined by using an estimate of partial volume probabilities at each voxel. After graph-cuts computation, the segmentation result was finely post-processed by morphological opening to reduce over- and under-segmented parts. Normalized hippocampal volumes were considered as ratios of intracranial volume (ICV).

#### Shape analysis

For the analysis of hippocampal shape, we used a shape modeling method based on a Laplacian surface deformation framework (28). A flowchart summarizing the overall workflow of the hippocampal shape analysis is provided in Supplementary Figure S1. The Laplacian surface deformation framework establishes vertex-wise correspondence between the template and individual hippocampal surface models while preserving local geometric relationships through non-rigid deformation. This enables quantitative comparison of local hippocampal shape variations across subjects (28). This shape modeling method is accessible as open-source software, “Deformable Template Model for Brain Analysis (DTM, version 1.2)”, and is available at: https://www.nitrc.org/projects/dtmframework/. Using binary hippocampal masks of healthy controls, a mean binary mask image was determined using an interactive and non-linear registration between them according to the method described by Heitz (29). The template surface model was determined by surface mesh construction and standard remeshing methods (30). To reconstruct the surface models of each individual, the template surface model was rigidly aligned to the binary masks of individual hippocampus using an iterative closest point algorithm (31). Next, each vertex of the template surface model was propagated into the target surface while their Laplacian coordinates were preserved using the shape modeling technique (28). The individual surface models of each healthy control and patient with INPH were aligned back to the template surface model using isotropic rescaling of shape models with intracranial volume and a generalized Procrustes analysis (GPA) to determine the shape deformity of each vertex from individual surface models with respect to the correspondent vertex in the template surface model (32). The local shape deformity at each vertex of individual shape models was determined as the signed Euclidean norm of the displacement vectors, projected on the vertex normal on the template surface model to determine the direction of local shape differences of individual hippocampus. The modeling and shape analysis processes were performed separately on the right and left hippocampus.

#### Statistical analysis

With the local shape deformity measurement, the following statistical analyses were used to measure regions of shape differences between the INPH patients and controls: (1) analysis of the mean deformity of patients with INPH and (2) the analysis of covariance (ANCOVA) with ICV as covariates to test the hypothesis that the local shape deformity can discriminate groups (28). We applied a false discovery rate (FDR) adjustment to the *p* values by ANCOVA to reduce the false positives. The hippocampal shape differences between the ^18^F-florbetaben-positive and ^18^F-florbetaben-negative INPH cases were further investigated using ANCOVA with sex, age, and ICV as covariates. To analyze the association between local shape deformity and gait performance, we used robust multilinear regression with iterative reweighted least squares and a bisquare weighting function. This analysis was performed in MATLAB using the robustfit function (28). R version 4.0.3 (https://www.r-project.org) was also employed for statistical analysis. Clinical characteristics, demographic data, and normalized hippocampal volumes were compared between the groups. Fisher’s exact and chi-square tests were used to compare categorical variables, and the Welch t test, Student’s t test, and Wilcoxon rank-sum test were employed to compare continuous variables. The differences in normalized hippocampal volume between ^18^F-florbetaben-positive and ^18^F-florbetaben-negative INPH cases were further investigated using ANCOVA, with sex and age as covariates. Pearson’s or Spearman’s correlations were employed to measure relationships between normalized hippocampal volumes and INPH gait disturbance.

## Results

In total, 48 INPH patients with a positive CSFTT response and 25 age- and sex-matched healthy controls were analyzed. Table 1 shows the demographic and clinical data of both INPH patients and healthy controls. There were no significant differences in sex and age between the groups. INPH patients demonstrated statistically lower K-MMSE scores than controls. Of the 48 INPH patients, 13 were shown to be amyloid-positive on florbetaben PET.

**Table 1:** Characteristics of participant samples used in analyses.

Characteristics	Controls vs. patients			INPH patients (*n* = 48)		
	Controls (*n* = 25)	INPH patients (*n* = 48)	*p*-value	INPH_A+_ (*n* = 13)	INPH_A-_(*n* = 35)	*p*-value
Gender, male	9 (36.0)	21 (43.8)	0.698	6 (46.2)	15 (42.9)	> 0.999
Age (year)	71.4 ± 3.0	72.6 ± 5.6	0.052	76.1 ± 3.1	71.4 ± 5.9	0.008
Apolipoprotein E ε4+/ε4−		6/24		4/3	2/21	0.016
K-MMSE	28.3 ± 1.6	20.6 ± 5.7	< 0.001	17.2 ± 8.3	21.9 ± 3.7	0.065
Timed Up and Go test		17.4 ± 6.8		18.4 ± 5.9	17.1 ± 7.1	0.215
10 meter walking test		16.9 ± 9.5		17.7 ± 9.8	16.6 ± 9.6	0.281
Normalized hippocampus volume						
Left	2.59 ± 0.27	2.01 ± 0.27	< 0.001	1.97 ± 0.30	2.02 ± 0.27	0.563*
Right	2.73 ± 0.24	2.13 ± 0.28	< 0.001	2.07 ± 0.27	2.15 ± 0.28	0.776*

Values are presented as number (%) or mean ± standard deviation. Group comparisons were performed using Fisher’s exact test or chi-square test for categorical variables and Welch’s *t*-test, Student’s *t*-test, or Wilcoxon rank-sum test for continuous variables, as appropriate. INPH, idiopathic normal-pressure hydrocephalus; INPH_A+_, amyloid-positive INPH subjects; INPH_A−,_ amyloid-negative INPH subjects; K-MMSE, Korean version of Mini-Mental State Examination.

^*^p-values were obtained using ANCOVA adjusted for age and sex.

### Volumetric analysis

As demonstrated in Table 1, the normalized hippocampal volumes of the INPH were smaller than those of healthy controls. However, within the INPH group, after adjusting for age and sex, the normalized hippocampal volume was not significantly different between amyloid-positive and amyloid-negative individuals.

In the INPH group, right normalized hippocampal volume was found to be negatively correlated with the TUG score (*r* = −0.289, *p* = 0.046) and 10-meter walking test score (*r* = −0.328, *p* = 0.023), whereas left normalized hippocampal volume was not significantly correlated with the TUG score (*r* = −0.264, *p* = 0.070) or the 10-meter walking test score (*r* = −0.222, *p* = 0.129).

### Shape analysis

Vertex-wise shape analysis revealed significant bilateral deformation in patients with INPH compared with healthy controls after FDR correction (Figure 1). Visual inspection of the group-level deformation patterns showed that both the left and right hippocampi exhibited a more elongated and narrowed morphology in INPH than in healthy controls. In both hemispheres, the middle and posterior hippocampal regions also showed upward bending, reflecting vertical displacement from the body to the tail and resulting in an overall upward-bowed appearance (Figure 1). Within the INPH group, after adjusting for age and sex, there was no significant difference in hippocampal shape between amyloid-positive and amyloid-negative individuals.

**Figure 1:**
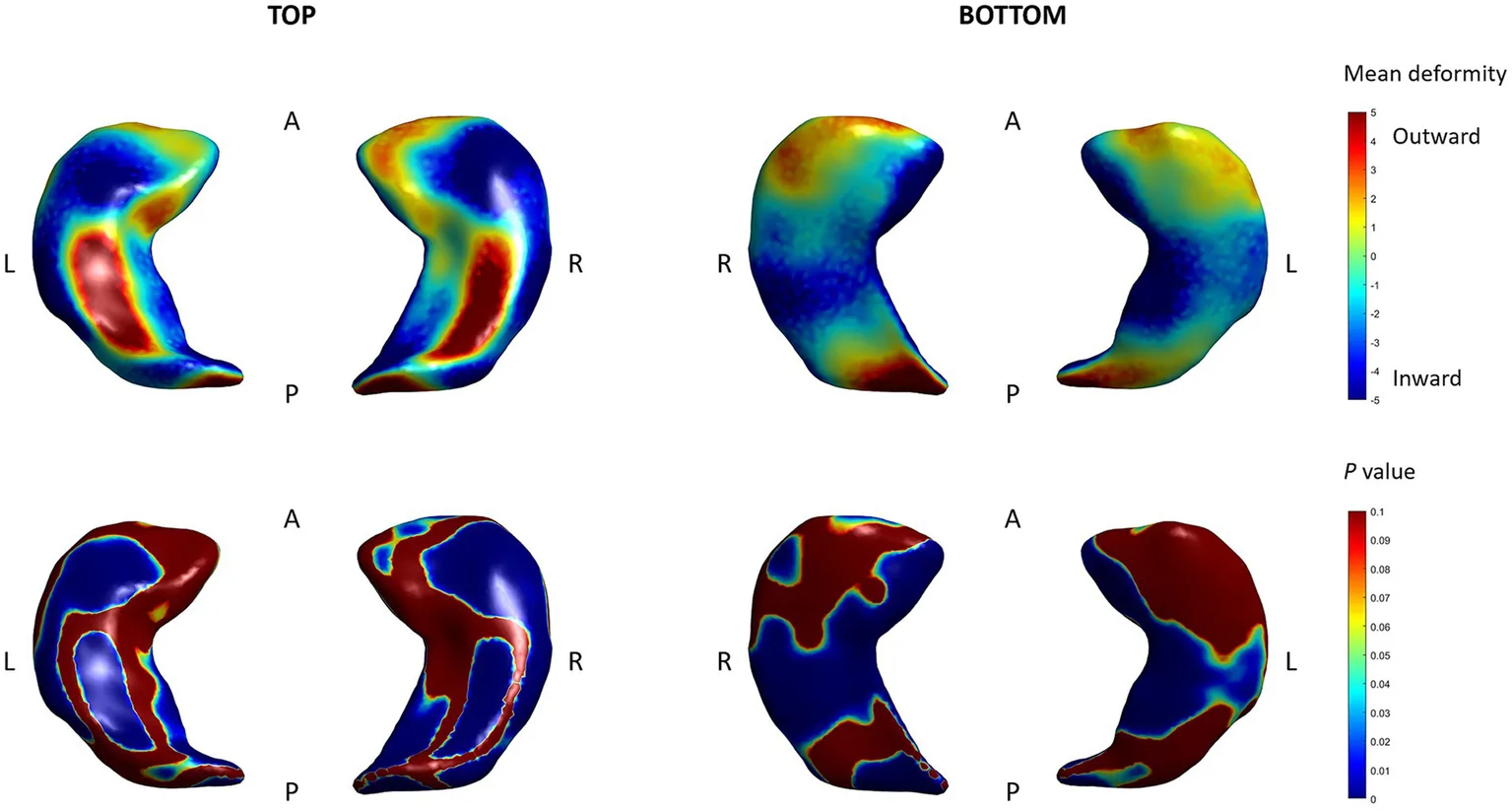
Vertex-wise comparison of hippocampal shape between patients with INPH and healthy controls. The upper row shows the magnitude and direction of mean local shape deformation in patients with INPH relative to healthy controls, displayed on the mean hippocampal surface of the healthy controls. Cooler colors indicate inward deformation, whereas warmer colors indicate outward deformation. The lower row shows the corresponding vertex-wise *p* values from the group comparison after adjustment for intracranial volume and false discovery rate (FDR) correction for multiple comparisons. Vertices with FDR-corrected *p* < 0.05 were considered statistically significant. L, left; R, right; A, anterior; P, posterior.

Significant associations were found between local shape deformity and both gait performance tests, to a greater degree on the right than on the left (Figures 2, 3). Bilateral inward deformations at the superomedial hippocampal tail and inferior hippocampal body were consistently associated with inferior performance across tests. Inward deformations at the right medial and lateral hippocampal head and right superior hippocampal body were also consistently associated with worse performance across tests.

**Figure 2:**
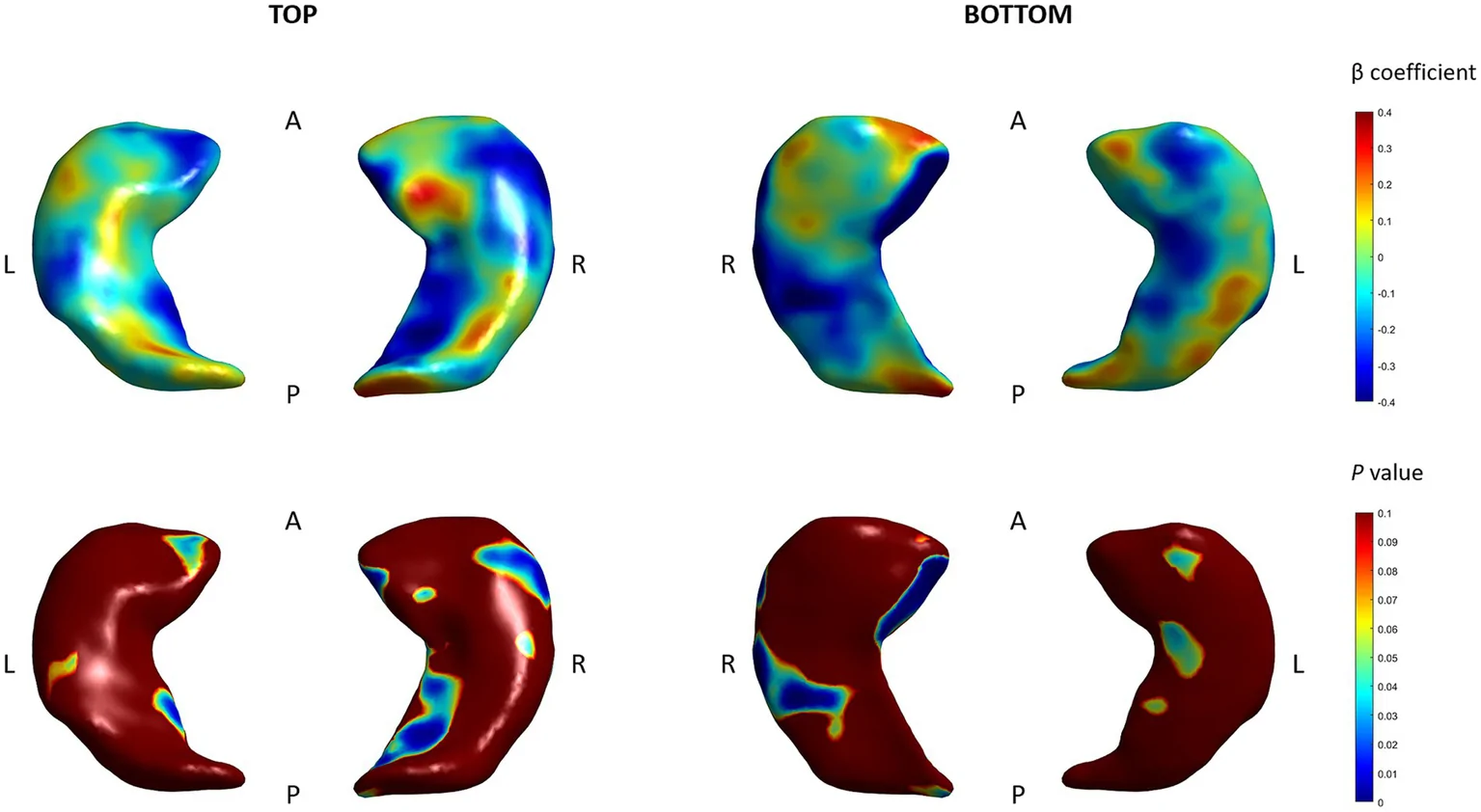
Vertex-wise association between hippocampal shape deformity and Timed Up and Go test scores in INPH patients. The upper row shows the unstandardized *β* coefficients derived from robust multilinear regression, representing the strength and direction of the association. The *p*-values presented are corrected for multiple testing with the false discovery rate (FDR) method (lower row). L, left; R, right; A, anterior; P, posterior.

**Figure 3:**
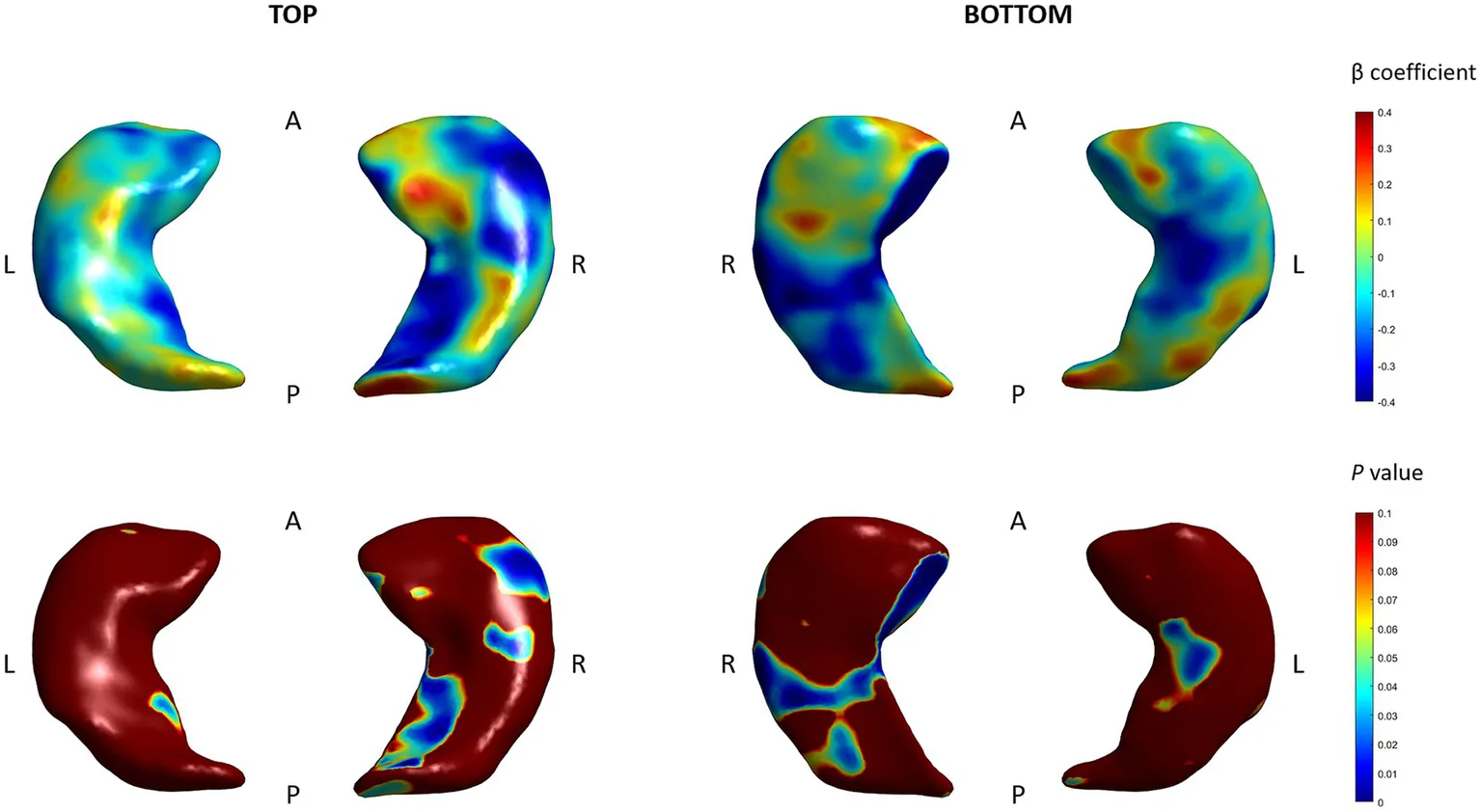
Vertex-wise association between hippocampal shape deformity and 10-meter walking test scores in INPH patients. The upper row shows the unstandardized *β* coefficients derived from robust multilinear regression, representing the strength and direction of the association. The *p* values presented are corrected for multiple testing with the false discovery rate (FDR) method (lower row). L, left; R, right; A, anterior; P, posterior.

## Discussion

Previous studies have reported inconsistent findings regarding hippocampal structural abnormalities in INPH. Importantly, volumetric measurements alone may not distinguish neurodegenerative tissue loss from morphological deformation associated with hydrocephalus (33). Moreover, the extent to which concomitant amyloid pathology contributes to hippocampal structural abnormalities in INPH remains unclear. These unresolved issues highlight the need to characterize hippocampal morphology beyond conventional volumetric measures and to examine its associations with amyloid pathology and the clinical manifestations of INPH.

Compared with age- and sex-matched healthy controls, INPH patients showed significantly smaller hippocampal volumes, and both hippocampi of INPH patients were narrower and more elongated, together with an upward bend of the posterior part of the hippocampus. Additionally, in INPH patients, right hippocampal volumes significantly correlated with gait dysfunction, as measured by the TUG and 10-meter walking test, and bilateral inward deformations at the superomedial hippocampal tail and inferior hippocampal body were consistently associated with worse performance across tests to a greater degree on the right than on the left.

Our first main result was that, in comparison to healthy controls, INPH patients had smaller hippocampal volumes, and both hippocampi were narrower and more elongated, together with an upward bend of the posterior part of the hippocampus. One potential mechanism underlying these morphological changes may involve the anatomical relationship between the hippocampus and the temporal horn of the lateral ventricle. Previous studies have demonstrated enlargement of the temporal horns in patients with INPH compared with controls (34). The temporal horn of the lateral ventricle forms the roof of the hippocampus, and the hippocampus is partially enclosed by the temporal horn (35, 36). In INPH, lateral ventricular enlargement may compress adjacent brain structures. For example, simultaneous expansion of the ventricles and Sylvian fissures is accompanied by compression of the high-convexity and medial subarachnoid spaces, a characteristic pattern of CSF distribution known as disproportionately enlarged subarachnoid-space hydrocephalus (DESH) (37). Furthermore, the hippocampus is closely related to the temporal horn superolaterally (36, 38), while the hippocampal head bulges into the temporal horn and the hippocampal tail occupies the medial part of the floor of the lateral ventricular atrium (39). Taken together, these anatomical relationships and the reported temporal horn enlargement in INPH suggest that ventricular enlargement may contribute to the characteristic pattern of hippocampal deformation observed in INPH. However, the present study did not directly assess the relationship between ventricular enlargement and hippocampal deformation; therefore, a causal relationship cannot be established from our findings.

Our second main result was that, in the INPH group, right hippocampal volumes correlated with gait dysfunction, as measured by the TUG and 10-meter walking test, and bilateral inward deformations at the superomedial hippocampal tail and inferior hippocampal body were consistently associated with worse performance across tests to a greater degree on the right than on the left. As a possible explanation for this result, we might speculate as follows. First, hippocampal volume, integrity, and metabolism are known to be associated with gait control in the elderly, especially in the right side rather than the left side (40–44). Second, it was reported that spatial navigation, visuospatial processing, and gait are related to each other (45–49). Third, both the subiculum and the presubiculum are known to play very important roles in spatial navigation (50–52). Considering all the aforementioned information, we can hypothesize that deficits in subiculum (inferior portions of hippocampus) and presubiculum (medial portions of hippocampus) may be involved in the pathogenesis of gait disturbance in INPH. However, in our study, it is difficult to precisely locate the inward deformations with respect to hippocampal subfields, and future studies are warranted to better investigate this aspect.

Interestingly, in our INPH patients, there were no significant differences in hippocampal volume and shape between the groups with positive and negative amyloid PET scans. Alzheimer disease (AD), the most common form of late-life dementia, is characterized by abnormal deposits of neurofibrillary tangles of τ protein and plaques of β-amyloid (Aβ) protein in the brain, eventually leading to neurodegeneration and cognitive decline. The accumulation of Aβ in the brain is believed to be a key factor in the development of AD (53). Amyloid pathology can also be found in cognitively unimpaired older adults (ie, probably preclinical stage of AD) and in patients with other neurodegenerative disorders (54). And it is well-known that amyloid PET can detect cerebral Aβ deposition with precision, has good specificity for AD neuropathology, and can also inform on the presence of contributing amyloid copathology in other diseases (54). It is of great interest to elucidate the relationship between hippocampal atrophy and Aβ load, to understand whether amyloid deposition may conduct to synaptic and neuronal degeneration as postulated by the amyloid hypothesis (55). It was reported that cognitively normal elderly individuals with a positive amyloid PET scan showed smaller hippocampal volumes relative to those with a negative amyloid PET scan (56). And the hippocampus is known to be affected early in AD (56). However, the evidence is mixed on the association of brain volumes and Aβ load in elderly subjects without clear cognitive impairment: Some studies have found no associations (53, 57). This inconsistency may be related to the stage of AD pathology of the samples (53). Based on our results, we can cautiously hypothesize that lateral ventricle enlargement plays a more important role in hippocampal changes in INPH than accompanying AD pathology.

INPH patients were selected in sequence from our registry. To limit possible bias, we employed grading scales considered objective before and after the CSFTT (rather than using subjective testimony by patients or their caregivers). Nevertheless, this study had some limitations. The first limitation was that this study did not include INPH patients with a negative response on the CSFTT. This was to increase the certainty of the INPH diagnosis, as INPH patients with a negative response on the CSFTT have a greater probability of having other neurological comorbidities (58). A second limitation was that pathologic tau biomarkers were not measured in our study. Consequently, AD staging could not be confirmed in our INPH patients. AD biomarkers were grouped as pathologic tau, Aβ deposition, and neurodegeneration (59). Aβ and pathologic tau biomarkers show specific neuropathologic changes that determine AD (59). Staging can be done by combining information from the three biomarker groups. In this case, a more advanced pathologic stage is associated with more abnormal biomarker groups (59). However, amyloid brain PET can exclude significant amyloid deposition and thus AD (60). Additionally, in our study, we believe that there might be merit in utilizing three-dimensional, surface-based, morphometric analysis of the hippocampus along with amyloid PET results in INPH patients. The vertex-wise shape analysis identifies localized morphological differences but does not provide a single global statistical measure of overall hippocampal shape. Therefore, descriptions of overall morphology, such as elongation and narrowing, should be interpreted as qualitative features of the observed deformation patterns rather than as independently tested global shape measures. Furthermore, ventricular and temporal horn volumes were not quantitatively assessed in the present study, and their direct relationship with hippocampal deformation warrants further investigation.

We found reduced hippocampal volumes in INPH patients as compared to controls. Unique patterns of hippocampal shape changes were also demonstrated in INPH patients. In INPH patients, both hippocampi were found to be narrower and more elongated, with an upward bend observed in the posterior part of the hippocampus, regardless of amyloid pathology. Moreover, the correlation between hippocampal structure and gait disturbance, which was more pronounced on the right side, suggests a possible role of the hippocampus in the control of gait and balance in INPH.

## Data Availability

The raw data supporting the conclusions of this article will be made available by the authors, without undue reservation.
